# Allergy Diagnosis in India: Current and Future Trends

**DOI:** 10.7759/cureus.112369

**Published:** 2026-07-09

**Authors:** Sitesh R Roy, Neeraj Gupta, Alap Christy, Aratrika Das, Krishna Mohan, Saibal Moitra, Prashant Nag, Karthik Nagaraju, Kasyapi Nagaraju, Sowmya Nagarajan, Reena Nakra, Suresh Natarajan, Mohit Poddar, Vikram S Patra, Roohi Rasool, Dhanesh Volvoikar, Khyati Doshi

**Affiliations:** 1 Allergy and Immunology, Dr. Roy Health Solutions Clinic, Mumbai, IND; 2 Pediatrics, Sir Ganga Ram Hospital, New Delhi, IND; 3 Clinical Chemistry, Metropolis Healthcare Limited, Mumbai, IND; 4 Pulmonology, Kolkata Chest and Allergy Centre, Kolkata, IND; 5 Pediatrics, Government Taluk Hospital, Malappuram, IND; 6 Allergy and Immunology, Apollo Multispeciality Hospitals, Kolkata, IND; 7 Diagnostics, Tata 1mg, Gurugram, IND; 8 Pulmonology and Allergy, VN Allergy and Asthma Research Centre, Chennai, IND; 9 Rhinology and Pediatric Allergy, VN Allergy and Asthma Research Centre, Chennai, IND; 10 Allergy and Immunology, Sanjeevini Specialist Clinic and Mallige Hospitals, Bengaluru, IND; 11 AI-Driven Diagnostics, Dr Lal PathLabs Ltd., Noida, IND; 12 Pediatric Allergy, Apollo Children's Hospitals, Chennai, IND; 13 Pediatrics, National Institute of Medical Sciences and Research, Jaipur, IND; 14 Pediatrics, Apollo Hospitals, Navi Mumbai, IND; 15 Immunology, Sher-i-Kashmir Institute of Medical Sciences, Srinagar, IND; 16 Pediatrics, Healthway Hospital, Alto Porvorim, IND; 17 Clinical Marketing, Thermo Fisher Scientific India Pvt. Ltd., Mumbai, IND

**Keywords:** allergic rhinitis, allergy, ige, india, in vitro diagnostics

## Abstract

Allergies, particularly allergic rhinitis (AR), atopic dermatitis (AD), and food allergies, are highly prevalent in India, especially among children. However, the true prevalence is likely underestimated, highlighting the need for improved diagnosis and management. This review highlights current allergy diagnostic practices in India and the associated challenges. Accurate diagnosis requires a comprehensive, allergy-focused patient history, correlated with appropriate in vivo and/or in vitro diagnostic test results. This necessitates awareness among healthcare professionals about allergies as a disease and the timely referral of patients to allergy specialists. However, ambiguities in diagnostic techniques, limited access to advanced diagnostic tools, high associated costs, fragmented allergy services, and evolving allergen profiles influenced by environmental and lifestyle changes present significant barriers to diagnosis. Addressing gaps in accessibility, affordability, and awareness is essential for establishing a robust framework for accurate diagnosis, ultimately improving patient outcomes and quality of life. The burden of allergic diseases in India remains underdiagnosed due to limited diagnostic capacity. Diagnosis is typically based on clinical history and tests, such as the skin prick test (SPT) and specific immunoglobulin E (IgE) measurement. However, challenges such as low awareness, unclear techniques, and limited access to standardized tools persist. Combining component-resolved diagnosis (CRD) with traditional tests can enhance accuracy and guide better treatment decisions.

## Introduction and background

Allergies are inflammatory conditions resulting from abnormal immune responses to specific proteins and glycoproteins known as allergens. These responses can trigger a range of symptoms, from mild hypersensitivity reactions to life-threatening responses [1,2]. Allergic diseases are common and affect millions of people, with their prevalence steadily rising [3,4]. In India, the prevalence of allergies is increasing across all ages and socioeconomic backgrounds, posing a major public health concern [5,6]. Currently, approximately 20-30% of the Indian population is affected by allergies caused by either aeroallergens or food allergens [5,6], with aeroallergens being the most predominant, leading to allergic rhinitis (AR) and asthma [7-10]. The burden of asthma in India exceeds that of HIV infection and tuberculosis [9]. The Global Asthma Network study from India shows comparable prevalence of current wheeze (adults: 3.30% vs. children: 3.34%) and doctor-diagnosed asthma (adults: 1.45% vs. children: 1.49%) between adults and children [11]. The incidence of AR in India is approximately 20% [5,12], which is higher than that reported in Western countries (children: 24.4% vs. 10.5%-13.3%; adults: 9.8% vs. 7.3%-7.7%) [7,8,13-15].

According to the Phase I study of the Global Asthma Network, conducted in India, nearly 2.5% of Indian children aged six to seven years and 3.48% of children aged 13-14 years have eczema [8]. A cross-sectional study in eastern India estimated the prevalence of contact dermatitis to be 4.38%. Based on occupational status, the prevalence was higher in urban than rural regions (24% vs. 12%, p<0.0001) [16]. A study conducted in allergy clinics across Maharashtra, Andhra Pradesh, and Delhi reported that *Dermatophagoides farinae *and cockroaches are the predominant allergens responsible for sensitization among children aged one to 14 years with asthma, AR, and atopic dermatitis (AD) [17]. Studies from southern and eastern India have shown high sensitization rates to* Dermatophagoides pteronyssinus, Acarus siro, Aspergillus fumigatus, Alternaria alternata, *and the pollens of *Cocos nucifera *and *Azadirachta indica* [18,19]. The high incidences of asthma, atopic allergies, and aeroallergen sensitization in these regions [5,18,19] are likely attributable to tropical and subtropical climates, high humidity, and seasonal variations [5].

Although food sensitization is common, the prevalence of food allergies in India is lower than that reported in Western countries (children: 0.14% vs. 5.8%-18.4%; adults: 1.2% vs. 6.2%-11.2%). However, several studies have reported high sensitization rates in Indian children to shrimp, sesame, buckwheat, wheat, melon, chickpeas, cumin, cabbage, eggplants, walnuts, and betel leaf [20-22], contributing to the increasing severity and prevalence of food allergies [5,21,23,24]. Drug allergies are a significant yet underreported concern in India, with an estimated prevalence of 5.6% [25,26]. Drug-induced allergic reactions can range from mild rashes to severe, life-threatening anaphylactic events. Antimicrobials, nonsteroidal anti-inflammatory drugs, and neuromuscular blockers are the most common triggers of anaphylaxis, particularly in perioperative settings [27], often complicating surgery or limiting therapeutic options, and potentially leading to prolonged hospitalization or fatal outcomes. Furthermore, approximately 30% of the Indian subcontinent is affected by insect allergies [11]. Patients with insect sting allergies can mainly develop urticarial symptoms, while insect bite allergies are primarily manifested as bronchial asthma. A study from eastern India reported higher sensitization rates for Hymenoptera insects (common black ant, fire ant, and honeybee) than the non-Hymenoptera group (mosquito, American cockroach, house fly) [28].

Given these risks, a timely and precise allergy diagnosis is critical. Proper diagnosis, based on an allergy-focused clinical history, the onset and periodicity of symptoms, and appropriate tests, is crucial for treatment selection [3,29]. The first step in treating an allergy is its accurate diagnosis. Most symptoms, including mild cutaneous or respiratory reactions, can be inconsistent and may overlap with existing comorbid conditions, leading to misdiagnosis, delayed treatments, and a reduced quality of life [30-33]. If left untreated, allergies can sometimes lead to anaphylaxis, affecting multiple organ systems. Therefore, early and accurate diagnosis of allergies can prevent future fatal reactions and disease progression, and improve patient outcomes [3]. This review details the allergy diagnostic tests currently used in India, highlighting the associated challenges and focusing on the emerging role of molecular diagnostics.

## Review

Methodology

Scholarly papers published in English from January 2000 to January 2024 were searched using PubMed and Google Scholar databases using search terms and medical subject headings (MeSH). The search terms included "allergy," "hypersensitivity," "prevalence," "India," “skin prick test," “skin tests," “in vitro testing," “specific IgE," “oral food challenge," "IgE," and "guideline." After eliminating the duplicates, the titles and abstracts were assessed, and publications with full texts that met the inclusion criteria were further evaluated.

Knowledge and awareness of allergy symptoms among healthcare professionals (HCPs) and patients

Allergy or hypersensitivity reactions can be classified as immunoglobulin E (IgE)-mediated or non-IgE-mediated [29]. IgE-mediated reactions can affect one or multiple organs, leading to cutaneous, oral, respiratory, gastrointestinal, cardiovascular, and neurological symptoms, which significantly impact patients’ quality of life [1,30,34]. Allergies may present with similar or distinct symptoms that occasionally overlap with other conditions, complicating diagnosis [1]. In children, AR is frequently undiagnosed or misdiagnosed as recurrent colds due to similar symptoms such as nasal congestion, sneezing, and cough. When cough is the primary symptom, particularly at night, AR may be misdiagnosed as “cough-variant” asthma. Additionally, perennial AR symptoms can mimic those of chronic sinusitis, recurrent upper respiratory infections, or vasomotor rhinitis [35]. Cough and dyspnea are common symptoms of both asthma and chronic obstructive pulmonary disease [36]. In elderly patients, exertional symptoms of asthma may be masked by the presence of cardiac or rheumatic diseases [33].

Food allergies are another significant concern. Children avoiding all forms of milk due to food allergies often exhibit reduced height and growth compared to their matched counterparts. Therefore, regular monitoring of growth is essential in these children to assess the risk of growth impairment [37]. Food allergy symptoms also overlap with gastrointestinal disorders, such as diarrhea, dysphagia, obstruction, vomiting, and malabsorption [32]. Appropriate diagnosis requires differentiating these conditions based on symptoms, mechanisms, and positive allergy test results [29,34,38]. Allergies can be missed by primary care physicians due to masked symptoms arising from comorbidities, symptom variability, inconsistency in manifestation and diagnostic testing, and lack of adequate training in allergy management [3,29,33]. Physicians should address the impact of food allergies and adopt measures through counseling, education, and psychotherapy to enhance patients’ quality of life [39].

Several Indian studies have shown that knowledge about allergies is limited among HCPs, patients, and caregivers [40-42]. This knowledge gap is attributed to insufficient training and a lack of appropriate educational tools. A study on patients with AR found that 68% of patients mistakenly believed AR to be contagious, indicating inadequate knowledge and negative attitudes that lead to poor practices and therapy adherence [42]. These challenges are further compounded by limited training in interpreting allergy tests, as allergy is not a specialized focus in the medical curriculum in India, and respiratory medicine is excluded from the core curriculum of the Bachelor of Medicine and Bachelor of Surgery degree. Allergy immunology is not recognized as a medical specialty in India, unlike in most other parts of the world, and institutions offering part-time diplomas, fellowships, or training programs are not recognized by the National Medical Commission [43]. Allergic diseases are managed by specialists in internal medicine, pulmonology, otorhinolaryngology, pediatrics, or dermatology, often causing delays in referrals to allergy specialists, thereby resulting in delays in diagnosis and management [40].

**Figure 1:**
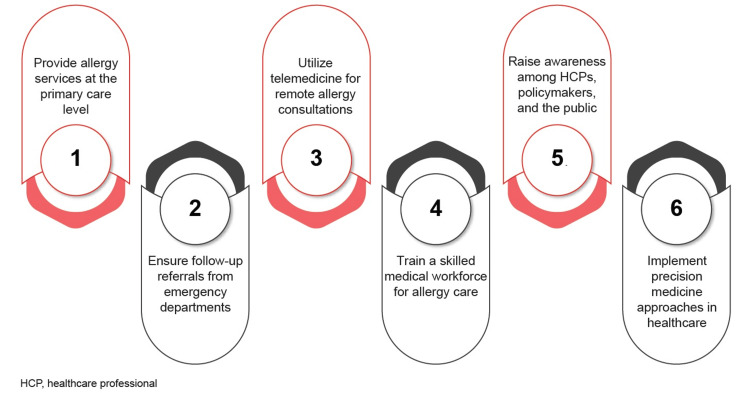
Steps to improve allergy diagnosis in India. The figure was created using Adobe Illustrator 2026 (Adobe Inc., San Jose, CA, USA).

Additionally, limited guidelines exist for allergy diagnosis in India; current guidelines based on data generated in Western countries may not apply to the Indian population [23]. Therefore, there is an unmet need for standardized guidelines and a referral-based treatment algorithm for allergy diagnosis in India (Figure 1).

Lack of India-specific diagnostic guidelines for allergy testing

The European Academy of Allergy and Clinical Immunology (EAACI) and the American Academy of Allergy, Asthma, and Immunology guidelines recommend a detailed allergy-focused medical history, followed by first-line in vivo tests, such as skin prick testing (SPT), and in vitro tests, such as specific IgE (sIgE) testing, for diagnosing allergies [3,29,44]. Although most clinicians in India follow these international guidelines in practice, there is a paucity of real-world studies validating their applicability to the Indian population. Evidence-based guidelines have recently been developed by the Indian College of Allergy, Asthma, and Applied Immunology; the South Asia Association of Allergy, Asthma, and Clinical Immunology; and the National Centre of Respiratory Allergy, Asthma, and Immunology for the diagnosis of allergic respiratory diseases in India [45].

Both in vivo (SPT, intradermal test, patch test, and allergen provocation test) and in vitro tests (serum IgE assays, total IgE and sIgE; and cell-based assays, such as the basophil activation test (BAT)) are currently used in India; however, some are used only for research purposes [33]. Among these, SPT remains the gold standard in vivo diagnostic method [46]. If SPT results are inconclusive, in vitro tests are ordered, followed by challenge tests that require specialized centers. The patch test can diagnose non-IgE-mediated allergies and delayed hypersensitivity reactions, but is considered redundant for respiratory allergies [3,47].

Current diagnostic practices and key challenges for allergy diagnosis in India

The first step in allergy diagnosis is obtaining a comprehensive, allergy-focused clinical history to identify allergy mechanisms, select appropriate allergens, and interpret test results (Table 1) [29,46]. A detailed history should include the time between allergen exposure and the onset of clinical symptoms; the number of affected body systems; frequency of symptoms (seasonal/perennial, diurnal); factors that worsen or alleviate symptoms; travel history; exposure to pets or insects; family history of atopy; and occupational history. Allergen selection should be based on clinical history and an individual cost-benefit analysis, but limited access to allergy services often delays diagnosis and treatment [46,48].

**Table 1:** Current global approaches for allergy diagnosis. ^#^such as ImmunoCAP; BAT: basophil activation test; CRD: component-resolved diagnosis; IDT: intradermal testing; IgE: immunoglobulin E; OFC: oral food challenge; SPT: skin prick test

Allergy tests	Mechanism detected
In vivo tests	
SPT	Type I, immediate, IgE-mediated allergy
IDT	Immediate IgE-mediated and delayed-type hypersensitivity
Patch test	Delayed-type, non-IgE-mediated
OFC	IgE-mediated allergy; sometimes delayed, non-IgE-mediated
In vitro tests	
Specific IgE^#^	IgE-mediated allergy
CRD	IgE-mediated allergy
BAT	IgE-mediated and non-IgE-mediated allergy

Skin Prick Test

This is considered the standard method for detecting IgE-mediated sensitization, especially for respiratory allergies [46,49]. SPT involves introducing an allergen extract into the epidermis using a disposable fine needle or lancet [50]. Although SPT using common aeroallergens is highly specific (80%) and sensitive (94.1%) in patients with/without AR [51], it is unsuitable for individuals at the extremes of age, pregnant women, those with skin conditions, patients with a history of anaphylaxis within the past four weeks, and those taking antihistamines. SPT requires an adequate area of healthy skin and patient cooperation, making it challenging in very young infants [49,52]. It also requires skilled personnel and a proper management setup. Although safe, anaphylaxis may rarely occur during SPT, with an incidence rate of 77 systemic reactions per 100,000 patients [52-54]. According to practice parameter guidelines, a wheal size >3 mm above the negative control is considered clinically accurate [55], although an Indian study demonstrated 100% sensitivity toward dust mites and cockroaches using a wheal size >4 mm [56]. The wheal size can vary depending on the pricking device, skill of the tester, and severity of the allergy [50]. In a study involving 69 patients with eczema, wheal sizes varied based on the expertise of the performer (dermatologist or nurse) and the type of device used (needles or lancets) [50].

SPT is also prone to false-positive and false-negative results due to non-standardized or improperly stored allergen extracts, inappropriate testing techniques (e.g., pricks made too close to one another, bleeding, or shallow penetration of the skin), abnormal immune reactions in the body and skin (such as cutaneous mastocytosis, dermatographism, and urticaria), and the use of antihistamines or poor diet [57-59]. This issue is highlighted in a study involving 52 patients with adverse drug reactions during anesthesia, which found that 7.69% showed false-positive SPT results for latex and 5.76% for neuromuscular blocking agents [57]. False positives can result from dermatographism or cross-reactive IgE antibodies, where one antibody is produced in response to a homologous protein and another comes from a different source. Such interactions may trigger allergies or be clinically irrelevant [60,61].

Another important concern with SPT is the quality of the allergen extracts used. A study evaluating the quality of commercially available dust mite extracts for SPT in India found that these extracts either lacked mite allergens entirely, contained only limited amounts, or included allergens from unrelated sources compared with globally approved standard allergen extracts [62].

Intradermal Testing

Intradermal testing may be considered in patients with a high suspicion of allergies but a negative SPT result, particularly in cases of adverse drug reactions [3]. This method is rapid, convenient, and reproducible for detecting drug hypersensitivity, commonly applied to penicillin, general and local anesthetic agents, tetanus toxoid, iodinated radiocontrast media, insulin, heterologous sera, collagen chymopapain, and others [63]. IDT can increase the chances of successful allergy immunotherapy (AIT) and successful treatment. A positive IDT, following negative SPT, can assist in detecting clinically relevant allergens for the diagnosis of AIT-responsive atopic disease [64]. Additionally, practice guidelines suggest that the IDT can be more sensitive than SPT due to the significantly greater amount of antigen that can be safely administered intradermally [65,66]. A prospective study involving 49 patients with adverse cutaneous drug reactions found the sensitivity of the IDT to be higher than SPT (49% vs. 73.5%, p<0.001) [67]. IDT is generally safe, with minimal adverse effects and a low risk of sensitization; however, anaphylactic reactions have been reported during this test [68,69]. Hence, resuscitative measures and epinephrine injections should be readily available. False-positive rates can be higher with intradermal tests compared with SPTs, particularly for mouse allergy [70]. Furthermore, intradermal skin tests have no predictive value in non-IgE-mediated reactions, such as serum sickness, hemolytic anemia, drug fever, interstitial nephritis, contact dermatitis, maculopapular exanthemata, or exfoliative dermatitis [63]. Additionally, it should be noted that intradermal testing may exhibit high positivity due to variations in the regional prevalence of aeroallergens, influenced by different geo-climatic conditions and the adaptation of local microbiota [71].

Patch Testing

Patch testing is another common method for detecting delayed hypersensitivity reactions, especially in patients with eczematous dermatitis or those suspected of having contact allergy [72]. However, patch tests do not detect IgE-mediated food or respiratory allergies [47,73]. An observational study of 111 cases of allergic contact dermatitis (ACD) reported a positive patch test in 69% of patients and identified potassium dichromate, parthenium, and para-phenylenediamine as the most common allergens triggering ACD [74]. Although the patch test is important for diagnosing the cause of ACD, results should be interpreted with caution. Additionally, varied interpretations of results and a lack of procedural homogeneity reduce its reliability across different observers. The absence of data from healthy controls for homemade preparations highlights the lack of standardization, resulting in higher rates of false positives and an increased risk of adverse reactions during testing [75].

Oral Food Challenge

The gold standard for diagnosing food allergies, including both IgE-mediated and non-IgE-mediated types, particularly nut allergies, is the oral food challenge (OFC) [76]. The OFC is especially valuable when conventional IgE-based testing yields inconclusive results, and it is useful for assessing tolerance to previously allergenic foods or determining the response threshold in children. However, conducting an OFC requires healthcare support, including trained physicians, nurses, and suitable hospital facilities, and it carries the risk of systemic reactions [77,78]. Given these shortcomings, specific IgE testing serves as a cornerstone of diagnostic evaluation, particularly when SPT is inconclusive or not feasible.

Specific IgE Testing

The respiratory guidelines of India, endorsed by the National Center of Respiratory Allergy, Asthma, and Immunology, consider total IgE a reliable marker for allergic bronchopulmonary aspergillosis but not for diagnosing respiratory allergy due to its low sensitivity and specificity [33]. The EAACI guidelines, however, recommend sIgE testing for allergy diagnosis because of its higher specificity compared to total IgE [29], since high total IgE levels may not always be related to atopy. Patients with medical conditions such as parasitic infestations, viral infections, and primary immunodeficiencies may have increased total IgE levels [79,80]. Smoking can also increase total IgE levels [81].

Currently, the lack of aerobiological data and limited molecular characterization of prevalent Indian allergens hinder IgE testing and the development of region-specific allergy panels [5,23]. Moreover, insufficient data on the sensitivity, specificity, positive/negative predictive values, and likelihood ratios of IgE testing in the Indian population make routine clinical use challenging for clinicians. The variability of non-standardized allergen extracts can make their detection challenging [29,33]. This is evident from a study involving 20 patients with bronchial asthma and/or AR, which reported a higher sensitivity of sIgE (>85%) for aeroallergens such as cockroaches, mosquitoes, and houseflies compared to SPT; however, the specificity was relatively low (37%-67%) [82]. Allergen extracts often consist of unpredictable blends of allergenic and nonallergenic components, which can vary and reduce sensitivity [3]. In another study involving 135 patients with asthma, AR, and a positive sIgE to food extracts (cut-off: 0.89-79 IU/mL), only 64 patients had food allergies as confirmed by OFC [59].

Additionally, the absence of an sIgE cut-off value hinders distinguishing between allergic and nonallergic reactions [56]. Studies demonstrate that sIgE levels are largely age-dependent, remaining very low in infancy, peaking around the age of 20, and then declining with age [83]. Given the limitations of SPT and IgE testing, Western countries are increasingly adopting precision medicine using component-resolved diagnosis (CRD) to identify IgE targeting specific allergen components [5,84].

Component-Resolved Diagnosis

This molecular diagnostic tool enables precise allergy identification and patient-tailored treatment by detecting specific epitopes through a comprehensive molecular profile analysis [84]. CRD is used when SPT or sIgE results are inconclusive or do not align with the patient’s medical history [29]. It is also feasible in cases where SPT cannot be performed and offers a less painful procedure, making it preferable for children. CRD demonstrates higher specificity than sIgE testing, especially in predicting primary and secondary nut allergies [76,85]. Notably, EAACI guidelines recommend performing CRD before conducting an OFC [29]. Additionally, CRD can accurately distinguish true sensitization from cross-reactivity and help identify triggers of anaphylaxis [84,86].

Based on CRD results, HCPs can stratify patients into high- or low-risk categories for systemic reactions and predict their tolerance to food or venom allergens [84]. Conducting CRD tests in patients with cow milk protein allergies can help assess the risk of severe reactions to both raw and thermally processed milk. Thus, CRD helps HCPs in making informed decisions regarding allergen challenges, immunotherapy, desensitization treatments, and treatment monitoring [87]. This aligns with an Italian study involving 651 children with pollen-related AR, which showed that CRD improved immunotherapy by identifying major components of grass pollens (*Phl p1* or *Phl p5*) [88]. CRD-based studies in India are limited. One study found that Indian patients with respiratory allergies and IgE sensitization to American cockroach (*Periplaneta americana*) and/or German cockroach (*Blattella germanica*) had component proteins Per a 3 (59%) and Per a 9 (57%) as major allergenic components [89].

**Figure 2:**
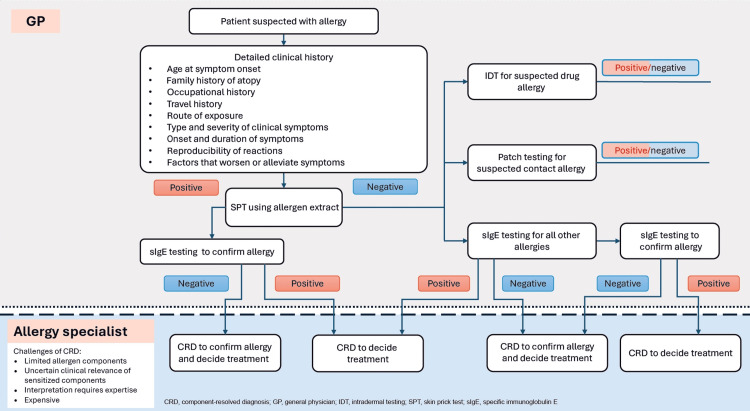
Flowchart outlining the journey of a patient suspected of having an allergy. The figure was created using Adobe Illustrator 2026 (Adobe Inc., San Jose, CA, USA).

CRD is a valuable approach for the diagnosis and management of food allergies; however, its results must be interpreted carefully to avoid unnecessary dietary restrictions and prescription of adrenaline auto-injectors [90]. Although CRD is not routinely used in India owing to its high costs, its superior accuracy, along with emerging India-specific allergen data, may support its future use in specific clinical scenarios, such as food allergy diagnosis and prognosis, anaphylaxis evaluation, optimized immunotherapy selection, and identification of relevant cross-reactivity (Figure 2) [5,90,91].

Emerging methods for allergy testing in India

BAT is still considered a research tool, particularly for nut allergies, as it has not yet been incorporated into standard clinical practice [76,92]. A preliminary study involving patients with asthma (unsensitized, sensitized to *Aspergillus*, or with allergic bronchopulmonary aspergillosis) and peanut sensitization revealed that basophil activation markers: CD63, CD193, and CD203c were unable to distinguish patients with and without allergic bronchopulmonary aspergillosis [93]. Additionally, limited donor availability, lack of standardized procedures, and higher costs further restrict its use [94].

Future directions

Allergy diagnosis in India is poised for effective management through enhanced infrastructure, advanced molecular diagnostics, integration of allergy training into medical education, and the establishment of clear referral pathways. Expanding access to standardized SPT in rural and smaller urban regions, along with improving the availability of advanced allergy diagnostics like CRD and BAT (Table 1) in urban centers, can help address existing gaps in care in order to align with global standards of allergy diagnostics. Introduction of continuing medical education and an allergy module in the medical curriculum can enhance allergy practices in India. Raising awareness among patients and policymakers, as well as the inclusion of subsidization for allergy diagnostics and treatment, can have a positive impact. There is a need for India-specific data on food and aeroallergens, their distribution, and the unique triggers of both IgE- and non-IgE-mediated reactions to better manage allergies in the Indian subcontinent. Furthermore, incorporating precision medicine approaches into healthcare practices would enhance allergy management.

## Conclusions

The prevalence of AD, AR, and food allergies in India is rising, but the actual burden remains underestimated, emphasizing the need for proper diagnosis and management. Accurate diagnosis relies heavily on clinical history and appropriate diagnostic tests (SPT, sIgE, CRD, and OFC), but several barriers hinder standardized allergy care in India. Key challenges include low awareness, ambiguity in current diagnostic techniques, and limited access to standardized extract tests and advanced diagnostic tools. Although SPT and sIgE testing are routinely used in clinical decision-making, their low specificity and inaccuracy make treatment decisions challenging. While CRD is an invaluable prognostic asset, high costs and infrastructural barriers limit its routine application in mainstream, resource-constrained Indian clinical practices. Combining SPT and IgE with CRD can enhance diagnostic accuracy, guiding decisions regarding allergen challenges and treatment selection.
